# Generation of intense magnetic wakes by relativistic laser pulses in plasma

**DOI:** 10.1038/s41598-023-28753-3

**Published:** 2023-01-30

**Authors:** Marcel Lamač, Uddhab Chaulagain, Jaroslav Nejdl, Sergey V. Bulanov

**Affiliations:** 1 ELI Beamlines Facility, The Extreme Light Infrastructure ERIC, Za Radnicí 835, Dolní Břežany, 25241 Czechia; 2 Faculty of Mathematics and Physics, Charles University, Ke Karlovu 3, Prague 2, 12116 Czechia; 3 Faculty of Nuclear Science and Physical Engineering, Czech Technical University in Prague, Břehová 7, Prague 1, 11519 Czechia; 4 Kansai Photon Science Institute, National Institutes for Quantum and Radiological Science and Technology, 8-1-7 Umemidai, Kizugawa-shi, 619-0215 Kyoto, Japan

**Keywords:** Laser-produced plasmas, Plasma-based accelerators

## Abstract

The emergence of petawatt lasers focused to relativistic intensities enables all-optical laboratory generation of intense magnetic fields in plasmas, which are of great interest due to their ubiquity in astrophysical phenomena. In this work, we study generation of spatially extended and long-lived intense magnetic fields. We show that such magnetic fields, scaling up to the gigagauss range, can be generated by interaction of petawatt laser pulses with relativistically underdense plasma. With three-dimensional particle-in-cell simulations we investigate generation of magnetic fields with strengths up to $10^{10}$ G and perform a large multi-parametric study of magnetic field in dependence on dimensionless laser amplitude $a_{0}$ and normalized plasma density $n_{e}/n_{c}$. The numerical results yield scaling laws that closely follow derived analytical result $B \propto \sqrt{a_{0}n_{e}/n_{c}}$, and further show a close match with previous experimental works. Furthermore, we show in three-dimensional geometry that the decay of the magnetic wake is governed by current filament bending instability, which develops similarly to von Kármán vortex street in its nonlinear stage.

## Introduction

Intense magnetic fields in plasmas are induced by fast charged particles, which can be accelerated by laser-plasma interaction. In the non-relativistic or mildly relativistic regime, magnetic field generation can be due to inverse Faraday effect, which can be understood as the exchange of angular momentum from light to charged particles, which leads to magnetization of plasma^1–5^. In the regime of relativistic intensities, nonlinear plasma response breaks the adiabaticity between plasma electrons and laser, which leads to non-reversible gain of energy and angular momentum and therefore quasistatic magnetic field generation^3,6,7^.

Laboratory generation of intense magnetic fields in plasmas attracts a lot of attention due to a broad range of applications^8^, such as magnetically enhanced fast-ignition fusion^9^, generation of collisionless shocks in magnetized plasma^10^, magnetically assisted ion acceleration^11–15^ or magnetic field reconnection research^16–19^. Of special interest is also generation of magnetic fields in the gigagauss (GG) range, where the magnetic cyclotron and atomic binding energies compete and atoms can deform into long rod shapes, leading to distinct atomic physics which occur in astrophysical objects like neutron stars and white dwarfs^20–22^. Even stronger magnetic fields are thought to exist in magnetars, where the magnetic fields cross the critical Schwinger field of quantum electrodynamics (QED) $B_{cr} = \frac{m_{e}^{2}c^{2}}{e\hbar }\approx 4.4\times 10^{13}$ G, where $m_{e}$ is the electron mass, *c* the speed of light, *e* the elementary charge and $\hbar$ the reduced Planck constant. In such magnetic fields atoms deform into narrow spindles and the vacuum itself becomes strongly polarized, which introduces a slew of additional physics of fundamental interest, such as vacuum birefringence, magnetic pair production, synergic synchrotron-Cherenkov radiation, photon splitting or scattering suppression^22–26^.

**Figure 1:**
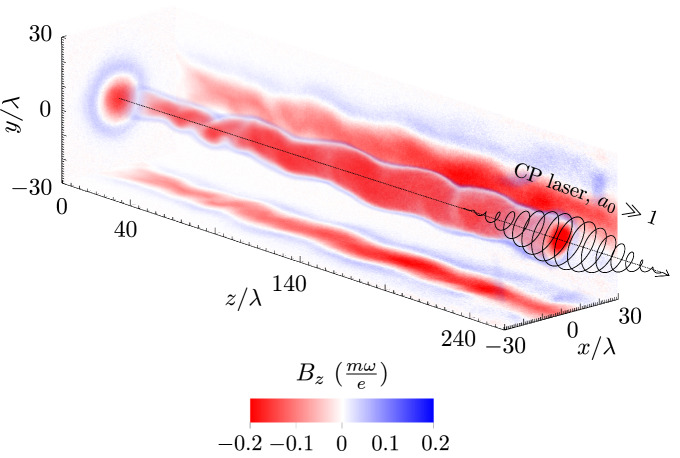
Schematic of intense magnetic wakefield generation by a relativistic laser in plasma. Magnetic fields generated by a relativistic right-hand circularly polarized relativistic laser pulse propagating in plasma. Axial component shown for relativistically underdense plasma where $n_{e}/n_{c} = 0.02$, $a_{0} = 80$ and pulse duration $\tau _{fwhm} = 11.7\,\, T$, where *T* is a laser period. Magnetic field given in units $\frac{m\omega }{e} = 110$ MG for $\uplambda = 1\,\,\upmu$m.

In recent decades, the process of laser-driven generation of magnetic fields has been investigated, in various schemes, theoretically^1–3,5,6,27–31^ and experimentally in underdense plasmas^4,32–34^ or solid targets^35^ up to relativistic intensities, $a_{0} \approx 1$^4,35^, where $a_{0} = \frac{eE_{0}}{m_{e}c\omega }$ is the dimensionless laser amplitude, $E_{0}$ the amplitude of the electric field and $\omega$ the laser angular frequency. Reaching $a_{0} \approx 1$ indicates a shift towards relativistic dynamics, where the electron momentum changes by more than $m_{e}c$ in a single laser cycle, leading to highly nonlinear plasma response, which can lead to geometrically non-trivial plasma currents. The advent of petawatt (PW) laser systems enables experimental investigation of magnetic field generation in plasma within ultra-relativistic regime $a_{0} \gg 1$, where magnetic fields reaching hundreds of megagauss (MG), or more, are expected^1,6,36^. These schemes often require disposable structured overdense targets or multiple laser beams to achieve gigagauss magnetic fields^15,37,38^. Underdense plasmas generated by a laser enable high-repetition rate, allow density tailoring and straightforward implementation of optical diagnostics^39^. While extensive research on magnetic field generation in underdense plasmas up to $a_{0} \approx 1$ has already been done, to the best of our knowledge, extended study involving multi-parametric numerical simulations focused on generation of long-lived intense magnetic fields is still lacking.

In this work, we study magnetic field generation with ultra-relativistic lasers in plasma over a broad range of parameters, from underdense to overcritical plasmas, through analytic theory and numerical simulations. We give an overview of the process of magnetic field generation in the relativistic $a_{0} \gg 1$ regime and specifically, being the experimentally most easily tunable parameters, we study dependency of the magnetic field on dimensionless laser amplitude $a_{0}$ and normalized electron density $n_{e}/n_{c}$ for relativistically underdense ($n_{e}/n_{c} < a_{0}$) plasmas, where $n_{e}$ is the electron density, $n_{c} = \frac{\omega ^{2}m_{e}\epsilon _{0}}{e^{2}}$ is the critical electron density and $\epsilon _{0}$ is the vacuum permittivity. We present a large numerical multi-parametric study, which shows magnetic field generation in plasmas with circularly polarized laser pulses up to $10^{10}$ G, and reveals scaling laws describing magnetic fields which can be also long-lived and macroscopically large, as shown in Fig. 1. We explore the temporal and spatial properties of the magnetic field and show in three-dimensional geometry that its decay is governed by bending instability of the electron current filament^40,41^, which leads to von Kármán antisymmetric electron vortex street development in its nonlinear stage. Furthermore, we show a close match of the obtained numerical scaling laws with magnetic fields observed in other experimental and theoretical works. Lastly, we discuss the possibility to use intense magnetic wakes to probe the physics of strong field QED.

## Results and discussion

### Analytical model for magnetic field strength

Let us consider a simple model where we assume efficient generation of a plasma channel by a circularly polarized laser pulse. This is valid for relativistically underdense plasmas when the laser waist size is given as $w_{0} \approx \sqrt{a_{0}}\frac{c}{\omega _{p}}$^14,42–44^, where $\omega _{p}^{2} = \frac{n_{e}e^{2}}{m_{e}\epsilon _{0}}$ is the electron plasma frequency. The magnetic field generated inside the plasma channel is given by Ampere’s law as

1 $$\begin{aligned} \nabla \times {\varvec{B}} = \mu _{0}{\varvec{j}}. \end{aligned}$$

where ${\varvec{j}}$ is the current density inside the plasma channel and $\upmu _{0}$ is the vacuum permeability. As it is shown in the following section, the magnetic field is induced primarily by helical motion of relativistic electrons. For simplicity, let us assume that the longitudinal and transverse velocity components of the electrons are equal and the electron speed approaches speed of light, $v_{i} \approx \frac{c}{\sqrt{3}}$. The current density components are then $j_{i} \approx -en_{e}\frac{c}{\sqrt{3}}$. Since the transverse size of the magnetic field corresponds to the transverse size of the plasma channel *l*, Eq. (1) becomes $B = \mu _{0}jl$. As mentioned above, in the case of a laser propagating in relativistically underdense plasma, a channel is generated with $l \approx \sqrt{a_{0}}\frac{c}{\omega _{p}}$, which gives us for the strength of the magnetic field components

2 $$\begin{aligned} B = \frac{1}{\sqrt{3}}\frac{m_{e}\omega }{e}\sqrt{\frac{a_{0}n_{e}}{n_{c}}}. \end{aligned}$$

We note that for efficient magnetic field generation the laser pulse must be short enough to prevent complete plasma cavitation, $\tau < \omega _{i}^{-1}$, where $\omega _{i}^{2} = \frac{n_{i}(Ze)^{2}}{m_{i}\epsilon _{0}}$ is the ion plasma frequency, where *Z* is the ion charge number, $n_{i}$ is the ion density and $m_{i}$ is the ion mass, otherwise the ions will be pushed out by the combined electrostatic and ponderomotive forces before any electrons pushed out are able to return and establish strong current inside the plasma channel through Weibel instability. We further note that laser waists with size $w_{0} > l$ are less efficient for magnetic field generation. This is due to the onset of laser filamentation instability, which limits the length scale of the plasma channel, and therefore the scale of magnetic field, to $l \approx \sqrt{a_{0}}\frac{c}{\omega _{p}}$^14,44^. We can recast Eq. (2) in practical units, such as the laser power *P*, since from the matching condition we have $a_{0} = 2(\frac{P}{P_{c}})^{1/3}$^43^, where $P_{c} = \frac{8\pi m^{2}c^{5}\epsilon _{0}}{e^{2}}\frac{\omega ^{2}}{\omega _{p}^{2}}$ is the self-focusing critical power, and therefore Eq. (2) becomes in practical units

3 $$\begin{aligned} B\,\,[\text {GG}] \approx 0.1\times (\uplambda \,\,[\mu \text {m}])^{1/3}(P\,\,[\text {PW}])^{1/6} (n_{e}\,\,[10^{20}\text { cm}^{-3}])^{2/3}. \end{aligned}$$

Let us now consider a technologically feasible scenario where we focus a laser with power $P = 1$ PW and central wavelength $\uplambda = 1\,\,\upmu$m into a spot matched to plasma with density $n_{e} = 10^{21}\text { cm}^{-3}$, which therefore corresponds to $a_{0} \approx 74.4$. According to either Eqs. (2) or (3), this generates quasistatic magnetic field with strength $B \approx 0.5$ GG, indicating the potential for intense magnetic field generation in relativistically underdense plasmas. We proceed towards quantitative investigation of this potential in the next section.

### Intense magnetic wake generation in underdense plasma by relativistic laser pulse

To self-consistently study magnetic field generation by ultrarelativistic laser in underdense plasma, we have performed a series of numerical simulations in three-dimensional (3D) Cartesian geometry (see Methods for simulation details). In this section we illustrate the mechanism of magnetic field generation by a relativistic laser pulse.

The laser field is defined as a right-hand circularly polarized laser propagating along the z-axis defined with normalized laser amplitude $a_{0} = 80$ and angular frequency $\omega = 2\pi c/\uplambda$, where $\uplambda = 1\,\,\upmu$m is the wavelength of the laser in vacuum. The circular polarization is chosen because we are interested not only in azimuthal magnetic fields, but also in axial magnetic fields, which are not generated for linear polarization and zero orbital angular momentum of the laser pulse, since in such a case there can be no longitudinal angular momentum transferred to plasma^1,2,7,37,45^. The laser pulse has a Gaussian spatial and temporal envelope with full width at half maximum pulse duration $\tau = 11.7\,\,T$, where $T = \uplambda / c$ is the laser cycle period. Such laser pulse duration is chosen to satisfy conditions $\omega _{p}^{-1}< \tau < \omega _{i}^{-1}$, which prevents complete ion cavitation before electrons manage to return inside the channel and establish helical currents. To prevent laser filamentation and enable efficient magnetic field generation, we match the beam waist of the focused laser to the plasma channel transverse size given by $w_{0} = \sqrt{a_{0}}\frac{c}{\omega _{p}}$. The target is composed of helium atoms with density $n_{He} = n_{e}/2$, where the normalized electron plasma density is established as $n_{e}/n_{c} = 0.07$ after barrier suppression ionization.

**Figure 2:**
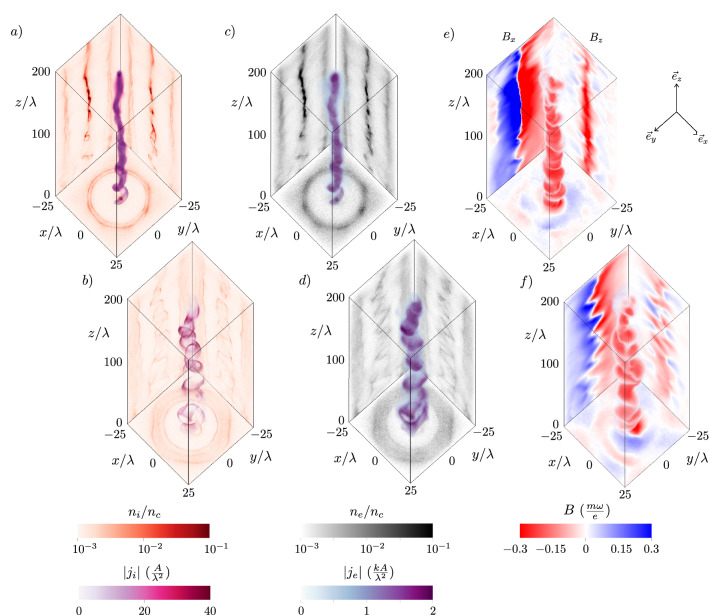
Evolution of quasistatic magnetic fields and plasma currents generated by ultrarelativistic laser. Ion current density magnitude $|j_{i}|$ (volumetric) and normalized ion density $n_{i}/n_{c}$ (central planes) at simulation time $360\,\,T$ (**a**) and $600\,\,T$ (**b**). Electron current density magnitude $|j_{e}|$ (volumetric) with normalized electron density $n_{e}/n_{c}$ (central planes) at simulation time $360\,\,T$ (**c**) and $600\,\,T$ (**d**). Axial magnetic field $B_{z}$ (volumetric, x-y and x-z central planes) and transverse magnetic field component $B_{x}$ (y-z central plane), which corresponds to the azimuthal magnetic field $B_{\phi }$, at simulation time $360\,\,T$ (**e**) and $600\,\,T$ (**f**).

The circularly polarized laser pulse enters the gas target at $t = 0$, accelerates electrons and leaves the simulation with highest energy electrons at $t \approx 360\,\,T$, leaving a plasma channel in its wake. During this time interval, the attraction of laser-accelerated plasma-wake electron currents flowing in the direction of the laser pulse propagation, in combination with the repulsion from the return currents flowing along the channel periphery, leads to on-axis current filament coalescence. This coalescence leads to current density distribution anisotropy, and therefore magnetic field development, according to the Weibel instability with a characteristic transverse scale $\sqrt{a_{0}}\frac{c}{\omega _{p}}$ and a maximum growth rate given by $\Gamma _{We} = \omega _{p}/\sqrt{a_{0}}$^46,47^. Figure 2 shows at $t \approx 360\,\,T$ the volumetric plot of the magnitude of the ion (a) and electron (c) current density, $|j_{i}|$ and $|j_{e}|$, which are opposite in direction, as well as the central planes of respective normalized particle densities $n_{i}/n_{c}$ and $n_{e}/n_{c}$, which also show the coalescent particle filaments as well as plasma channel walls containing the electron return current sheets ensuring plasma charge neutrality. The saturated magnetic fields are shown in Fig. 2e, with a volumetric plot of the axial magnetic field $B_{z}$, with central planes of both the axial magnetic field $B_{z}$, as well as the $B_{x}$ component, which represents the magnitude of the azimuthal magnetic field $B_{\phi }$ in the y-z central plane. We draw attention to the large aspect ratio of the longitudinal and transverse coordinate axes, which reveals a very long region of intense magnetic wakefield, which is homogeneous over a length of 200 $\uplambda$, as was shown for different parameters in Fig. 1.

**Figure 3:**
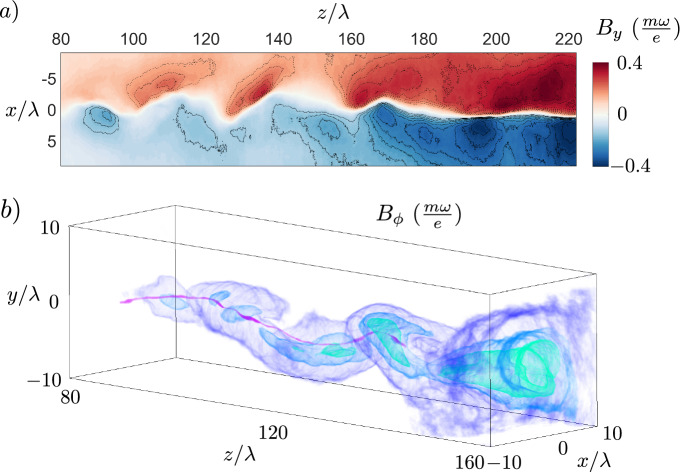
Bending instability of a current filament. (**a**) Magnetic field component $B_{y}$ (central x-z plane) with solid (dashed) isocontours showing electron fluid velocity streamlines indicating negative (positive) vorticity at $t = 378\,\,T$. (**b**) Volumetric isocontours of azimuthal magnetic field component for $B_{\phi } = 0.03, 0.18, 0.23, 0.28\,\,\frac{m\omega }{e}$ (purple, dark blue, light blue, green) showing the helical topology of the vortices at $t = 378\,\,T$.

Due to the nonlinearity of the plasma wavebreaking producing fast electron currents, the filament grows spatially inhomogeneous and develops a bending instability even in the case of linear polarization, as discussed in Ref.^40,41,47^, showing a transition from the transverse-symmetric current evolution in the case of linear plasma response ($a_{0} < 1$) discussed in previous works^28,29^. Figure 2b,d,f show the snapshots of the plasma evolution at $t = 600\,\,T$, where we see pronounced bending of the current filament in the late stage of the evolution. For initially irrotational plasma, the electron fluid vorticity is frozen into the magnetic field, since $\nabla \times {\varvec{p}} = e{\varvec{B}}$, where ${\varvec{p}}$ is the electron momentum, therefore the bending of the current filament leads to development of isolated electron fluid vortices. In two-dimensional geometry, this instability manifests as the bending instability of a current sheet^40^, which initially develops symmetric electron vortex pairs with vortex size of the order of or larger than the collisionless skin depth $c/\omega _{p}$ and with separation distance of the order of the plasma wavelength $\uplambda _{p} = 2\pi c/\omega _{p}$. The bending instability further separates these vortices into an antisymmetric electron vortex row similar to the von Kármán vortex street^48^. In Fig. 3a, we show a central slice through the transverse component of the magnetic field $B_{y}$, which shows the initial development of the bending instability and the emergence of antisymmetric vortex pairs, in agreement with two-dimensional results^40,41^. Fully three-dimensional topology of the vortices is shown in Figs. 3b and 2f, which show them following the helical bending of the current filament. Late stage of the evolution is shown in Fig. 2b,d,f, at which point the growth rate of the instability is slower and the antisymmetric von Kármán vortex row is well established. It can be seen in the $B_{x}$ component of the magnetic field in Fig. 2f corresponding to electron vorticity in the same direction.

**Figure 4:**
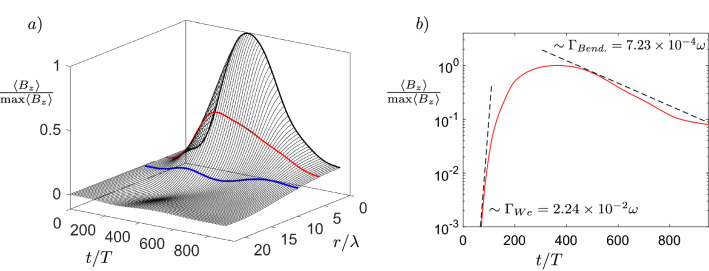
Temporal evolution of generated magnetic field. (**a**) Longitudinally averaged radial lineout of axial magnetic field with slices highlighted at radial distance $r/\uplambda = 0\text { (black)}, 4\text { (red)}, 8\text { (blue)}$. (**b**) Axial magnetic field for $r/\uplambda = 0$. max$\langle B_{z}\rangle = 0.3 \frac{m\omega }{e}$.

**Figure 5:**
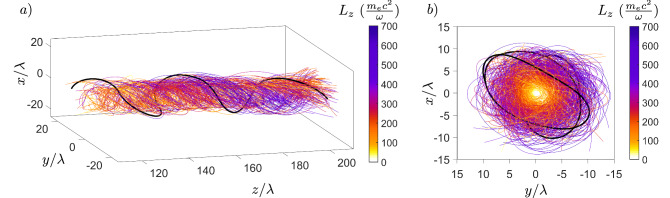
Electron trajectories generating axial and azimuthal magnetic fields. (**a**) Longitudinal angular momentum evolution with a z-direction view (**b**) shown in color, where $\frac{m_{e}c^{2}}{\omega } = 263776\,\,\hbar$ for $\uplambda = 1\,\,\upmu$m, which reveals the large absorption of right-handed photon spin angular momentum $S_{z} = +\hbar$ by electrons. The black curve highlights a single electron trajectory with angular momentum $L_{z} = 500\,\,\frac{m_{e}c^{2}}{\omega }$.

To quantify the temporal evolution of the quasistatic magnetic field, as well as the instability, over the whole region of interest, we define the longitudinally averaged radial lineout of the axial magnetic field as $\langle B_{z} \rangle (r) = \frac{1}{2\pi L}\int _{0}^{2\pi } \int _{0}^{L} B_{z}(r,\phi ,z)\,\,dz d\phi$. Temporal evolution of $\langle B_{z} \rangle (r)$ with $L = 200 \,\,\uplambda$ is shown in Fig. 4, where we see the growth of the electron Weibel instability between $t = 0$ and $t = 360\,\,T$ with maximum growth rate $\Gamma _{We} = \omega _{p}/\sqrt{a_{0}} \approx 2.24\times 10^{-2}\omega$, at the end of which it saturates as the magnetic energy density becomes comparable to the energy density in the electron flow $B_{sat.} \approx \frac{m\omega }{e}\sqrt{\frac{a_{0}n_{e}}{n_{c}}}$^46,47^ in accordance with Eq. (2). As the bending instability takes over the evolution at $t = 360\,\,T$, it leads to decay of the longitudinally averaged magnetic field. At the initial stage of the instability, the distances between the vortices are estimated as $5\,\,\upmu$m and $2.5\,\,\upmu$m in the horizontal and vertical direction respectively, with perturbation wavelength estimated as $20\,\,\upmu$m. Calculating the growth rate of the instability for the antisymmetrical vortex row from Eq. (5) in Ref.^40^, we get $\Gamma _{Bend.} \approx 7.27\times 10^{-4}\omega$, which matches the observed decay rate of the magnetic field. The bending instability starts to slow at the late nonlinear stage $t = 600\,\,T$, in accordance with results of Refs.^40,41^. The topology of the bending instability is similar to the drift-kink instability of the ion current filament discussed in Ref.^49^, however, the growth rate of the ion kink instability is at least an order of magnitude smaller. It is the faster electrons which are responsible for the topology and evolution of the magnetic field in our case, which validates the assumptions of the bending instability discussed in Refs.^40,41^. The full width at half maximum duration of the magnetic field is found to be $\tau _{B} \approx 390\,\,T$, which is in the picosecond range. This is up to two orders of magnitude longer than the duration of magnetic fields reported in other works^30,38^. Samples of helical trajectories of electrons carrying the longitudinal and transverse currents generating transverse and longitudinal magnetic fields respectively and evolution of their longitudinal angular momentum $L_{z}$ are shown in Fig. 5.

### Multi-parametric study in underdense plasma

To study the magnetic field strength dependence on normalized laser amplitude $a_{0}$ and normalized electron density $n_{e}/n_{c}$, we have carried out series of 3D PIC simulations. The parameter range of the PIC simulations (see Methods for details) corresponds to currently available laser systems and *f*-numbers of focusing optics capable of reaching the relativistic regime with $a_{0} \gg 1$. For $\uplambda = 1\,\,\upmu$m, the range of underdense plasma densities corresponds to $10^{18} - 10^{20}\text { cm}^{-3}$.

We measure the maximum cycle-averaged magnetic field strength of the longitudinal $B_{z}$ and azimuthal $B_{\phi }$ field component after the laser leaves the simulation. Performing nonlinear regression on the obtained data set yields the following numerical scaling laws for the strength of magnetic field components

4a $$\begin{aligned} \frac{eB_{z}}{m_{e}\omega }&= 0.22\left( \frac{n_{e}}{n_{c}}\right) ^{0.59}a_{0}^{0.56}, \end{aligned}$$

4b $$\begin{aligned} \frac{eB_{\phi }}{m_{e}\omega }&= 0.61\left( \frac{n_{e}}{n_{c}}\right) ^{0.65}a_{0}^{0.44}. \end{aligned}$$

**Figure 6:**
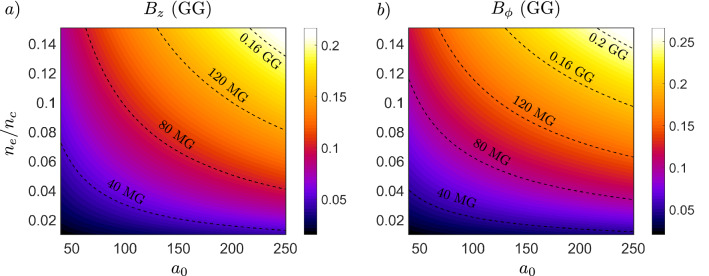
Numerical scaling laws for magnetic field components. Dependence of magnetic field components $B_{z}$ (**a**) and $B_{\phi }$ (**b**) on normalized electron density $n_{e}/n_{c}$ and normalized laser amplitude $a_{0} \gg 1$. For laser wavelength $\uplambda = 1\,\,\upmu$m.

We see a good correspondence between Eqs. (4) and Eq. (2), which validates the assumptions of the simple analytical model (2). We plot the magnetic fields given by Eq. (4) within the scanned parameter range in Fig. 6.

More interestingly, Eq. (4) fit well with magnetic fields measured in other works outside the studied parameter range. Picosecond-long axial magnetic field with magnetic field $B_{z} \approx 3.9$ MG generated in underdense plasma was experimentally measured in Ref.^4^ for $\uplambda = 1.054\,\,\upmu$m, $a_{0} = 2.5$ and $n_{e}/n_{c} = 0.02$. Plugging these values into Eq. (4a) gives axial field strength of $B_{z} \approx 3.8$ MG, showing a close correspondence, which is not present with analytical models presented therein that heavily underestimate the magnetic field strength, as discussed by the authors. Another experimental work^33^ observed axial magnetic field due to nonlinear inverse Faraday effect with $B_{z} \approx 1.5$ MG for $\uplambda = 1.06\,\,\upmu$m, $a_{0} = 9\times 10^{-3}$, $n_{e}/n_{c} \approx 1$ and for such values, Eq. (4a) gives $B_{z} \approx 1.6$ MG. Similarly, in the numerical work^15^, long-lived azimuthal magnetic fields of $B_{\phi } \approx 1$ GG can be seen generated in the wake of the main magnetic vortex trailing behind a laser propagating in dense plasma for $\uplambda = 0.8\,\,\upmu$m, $a_{0} = 80$ and $n_{e}/n_{c} = 3.32$. Plugging these values into the scaling law for the azimuthal field component Eq. (4b) gives $B_{\phi } \approx 1$ GG. This correspondence holds for all the other cases discussed there as well. These agreements show the applicability of the scaling laws (4), in order of magnitude, to a broader range of parameters in the underdense regime due to the dense data set of the multi-parametric study, which strenghtens their predictive potential.

### Extremely intense magnetic wake generation in dense plasma

**Figure 7:**
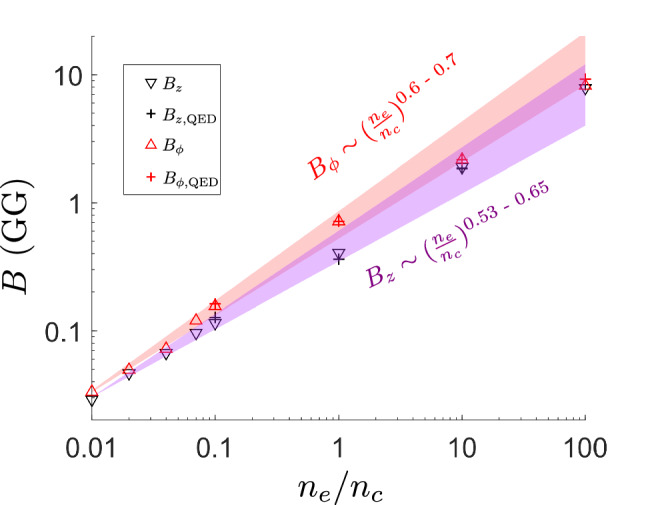
Magnetic field strength dependence on normalized electron density for ${a_{0} = 200}$. Magnetic field strength of components $B_{z}$ and $B_{\phi }$ measured from simulations with numerical scaling laws plotted within $95\%$ confidence bounds of electron density dependence power index obtained through nonlinear regression. Simulations were performed both with (crosses) and without (triangles) quantum electrodynamics effects for $n_{e}/n_{c} \ge 0.1$.

**Figure 8:**
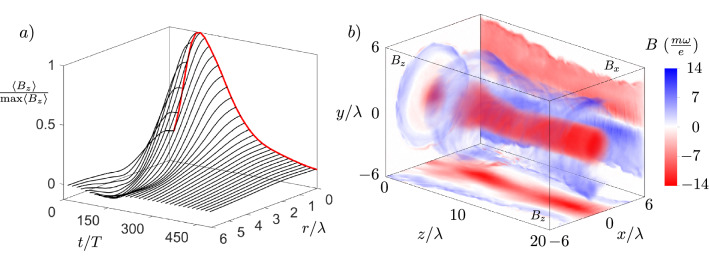
Extremely intense magnetic field generated for ${a_{0} = 200, n_{e}/n_{c} = 10}$. (**a**) Time evolution of longitudinally averaged ($L = 20\,\,\uplambda$) radial lineout of axial magnetic field. (**b**) Longitudinal (volumetric, x–y and x–z central planes) and transverse (y–z central plane) field components at magnetic field saturation time $t = 120\,\,T$.

To further evaluate the predictive potential of Eqs. (2) and (4) for higher plasma densities, we conduct additional simulations of magnetic field generation in dense targets for $$a_{0} = 200$$ (see Methods for details). Figure 7 shows the strength of the obtained magnetic fields corresponding to gas, liquid or solid, but relativistcally underdense ($$n_{e}/n_{c} < a_{0})$$ targets. Intense magnetic fields reaching up to $$\approx 10^{10}$$ G are found in accordance with Eqs. (2, 4). The temporal and spatial properties of the magnetic field with $$B_{z} \approx 1.8$$ GG and $$B_{\phi } \approx 2$$ GG generated for the case of $$n_{e}/n_{c} = 10$$ are shown in Fig. 8a,b, from which we see that the temporal duration of the magnetic field equals $$\tau _{B} \approx 165\,\,T$$, which is 0.5 picosecond for $$\uplambda = 1\,\,\upmu$$m, and the magnetic field extends homogeneously over a length of $$20\,\,\uplambda$$ at the time of Weibel instability saturation $$t = 120\,\,T$$.

Additionaly, we have performed simulations both with and without the inclusion of effects of QED as described in Ref.^50^. We have found that these processes, notably radiation friction, have negligible effect on magnetic field generation in the scanned parameter range, which is shown in Fig. 7. This is in accordance with the results of Ref.^36^, where it was discussed that energy dissipation through radiation friction is of the order of energy lost due to numerical error for $a_{0} \le 200$ for $n_{e}/n_{c} = 90$, which we now confirm for plasma densities in the range $n_{e}/n_{c} = 0.1 - 100$.

We proceed to consider the use of intense long-lived magnetic wakes generated by relativistic lasers for laboratory probing of strong field QED effects in magnetized plasma^23,24^. QED processes are essential in magnetized plasmas surrounding pulsars and especially magnetars^21,22,24,26^, and their transition probabilities generally increase with the Lorentz invariant parameter $\chi = \gamma (B/B_{cr})$, where $\gamma$ is the Lorentz factor of the electron interacting with magnetic field. The value of $\chi \approx 1$ indicates the quantum regime threshold, beyond which quantum effects cannot be neglected^23^. Sending relativistic electrons with $\gamma \approx 10^{3-4}$, which could be produced in the same optically synchronized setup through laser wakefield acceleration^51^, to a magnetic wake with $10^{10}$ G gives magnetic field in the electron rest frame as $B \approx B_{cr}$ and therefore the Lorentz invariant quantum parameter becomes $\chi \approx 1$. Recent experimental work with relativistic electrons accelerated up to $\gamma \approx 10^{5}$ verified the necessity for the use of quantum models of synchrotron radiation for $\chi \approx 1$ using a crystal as a target, which is limited to a field corresponding to $10^{8}$ G^52^. Electrons with $\gamma \approx 10^{5}$ would generate synergic synchrotron-Cherenkov radiation in tenuous plasma even with magnetic field $\approx 10^{3}$ G, as discussed in Ref.^24^. Interacting with extremely intense magnetic wakes reaching $10^{10}$ G, such electrons would experience $\chi \ge 10^{2}$, which would enable probing of strong field QED in magnetized plasmas close to the conjectured non-perturbative regime threshold $\alpha \chi ^{2/3} \approx 1$^53–55^, where $\alpha$ is the fine-structure constant. These considerations indicate the potential of laser generated intense magnetic wakes to serve as a test bench for strong field QED in magnetized plasmas over a broad range of quantum parameters $\chi$, which is possible due to the tunability of magnetic field strength through $a_{0}$ and $n_{e}/n_{c}$, as given by Eq. (2).

## Conclusion

We have investigated analytically and numerically generation of spatially extended, long-lived and intense magnetic fields by circularly polarized relativistic laser pulses in plasmas for the parameter range $a_{0} = 40 - 200$, $n_{e}/n_{c} = 0.01 - 100$. We show that circularly polarized relativistic laser pulse transfers angular momentum to plasma electrons, leading to development of strong helical currents through Weibel instability that sustain the longitudinal, as well as the transverse, component of the magnetic field. These currents can sustain long-lasting intense magnetic fields with strengths up to $10^{10}$ G inside the plasma channel. We have identified the magnetic field decay due to bending instability, which develops similarly to von Kármán vortex street in its nonlinear stage. Furthermore, we have performed a multi-parametric study of magnetic field strength dependence on $a_{0}$ and $n_{e}/n_{c}$ in relativistically underdense plasmas ($n_{e}/n_{c} < a_{0}$), which revealed numerical scaling laws (4) that follow the analytical result (2). We have found agreement between the scaling laws (4) and magnetic fields produced in other experimental and theoretical works, which further supports their predictive power over a broad range of parameters in the relativistically underdense regime. Finally, we envision interactions of relativistic electrons with studied intense magnetic wakes for probing of strong field quantum electrodynamics in magnetized plasmas.

Our results pave way towards generation of intense, tunable and long-lived magnetic fields in plasmas at various laboratory conditions, which lead to innumerable applications in plasma physics, fundamental physics or laboratory astrophysics.

## Methods

### Particle-in-cell simulations

We have performed numerical simulations in the full 3D Cartesian geometry with the relativistic, massively parallelized PIC code EPOCH^56^. Second order Yee scheme Maxwell solver and Higuera-Cary algorithm are used for the field and particle evolutions respectively. To reduce numerical dispersion in the second order scheme, Courant-Friedrichs-Lewy number was set to $c\Delta t/\Delta z = 0.99$. Third-order interpolation is employed to reduce grid heating. The size of the simulation box corresponding to the benchmark simulation results presented in Figs. 2, 3, 4 and 5 was set as $320\,\,\uplambda \times 50\,\,\uplambda \times 50\,\,\uplambda$ with grid size $4000 \times 360 \times 360$. The number of macro-particles per cell is 4, giving a total of $2\times 10^{9}$ macro-particles for helium ions and $4\times 10^{9}$ macro-particles for electrons. We use absorbing boundary conditions for both particles and fields. The simulation is evolved up to simulation time $t_{end} = 1500\,\,T$, which is long enough to see the full temporal evolution and decay of the magnetic field. Finally, as we are interested in the slow quasistatic evolution of the magnetic fields, all field variables were averaged every laser cycle as ${\bar{A}}= \frac{1}{T}\int _{0}^{T}A(t)\,\,dt$, where *T* is the laser cycle period. With this remark, we do not use the bar notation in the article.

For the multi-parametric study in underdense plasmas (Fig. 6), due to large computational demands of 3D simulations, we have reduced the number of macro-particles per cell to 2 and considered the ionised helium atoms as immobile. We have found that this approximation leads to negligible differences in generated magnetic field strengths, since the electron and ion current densities differ by two orders of magnitude (Fig. 2) and the Weibel instability of the electron current filaments is the main source of the magnetic field, as discussed in the article. We note that other properties of the magnetic field not investigated with the multi-parametric study, such as spatial and temporal homogeneity, are affected by ion immobility, as discussed elsewhere^31^. Since the laser spot size is changing for each data point according to $w_{0} = \sqrt{a_{0}}\frac{c}{\omega _{p}}$, the dimensions of the simulation box are decreased with decreasing $w_{0}$. The resolution of the grid is set for each case to resolve $\uplambda = \text {min}(\uplambda _{p},\uplambda _{L})$ with at least 10 samples, which assures sufficient resolution of the important wavelengths according to the Nyquist-Shannon sampling theorem. The study is performed over the span of following parameter values: $a_{0} = 40, 80, 120, 160, 200$ and $n_{e}/n_{c} = 0.01, 0.02, 0.04, 0.07, 0.1$.

Simulations of dense targets presented in Figs. 7 and 8 with mobile ions and also with and without QED effects were further performed for $a_{0} = 200$. The number of electron and ion particles per cell was chosen as 4 and 2 respectively. The plasma density range was selected as $n_{e}/n_{c} = 0.01, 0.04, 0.07, 0.1, 1, 10, 100$. In addition to third-order interpolation, current smoothing is turned on for $n_{e}/n_{c} \ge 1$ to ensure suppression of grid heating^56^. The resolution and size of the simulation box was selected according to the same criteria as in the multi-parametric study, e.g. for the lowest density $n_{e}/n_{c} = 0.01$, we set $\frac{\uplambda _{p}}{\Delta x,y} = 67, \frac{\uplambda _{p}}{\Delta z} = 125, \frac{\uplambda _{L}}{\Delta x,y} = 13$ and for the highest density $n_{e}/n_{c} = 100$, we set $\frac{\uplambda _{L}}{\Delta x,y,z} = 100, \frac{\uplambda _{p}}{\Delta x,y,z} = 10$. For $n_{e}/n_{c} \ge 10$, the waist of the laser is fixed to $1\,\,\upmu$m as the relativistic skin depth is smaller than laser wavelength and we do not consider tight focusing of the laser. This leads to energetically less efficient plasma channel and magnetic field generation due to laser filamentation, however, the Weibel saturated magnetic field is fixed by a choice of $a_{0}$ and $n_{e}/n_{c}$^44,46,47^.

We have performed convergence tests that involved doubling the number of particles and increasing transverse resolution which produced minimal differences in peak magnetic fields.

## Data Availability

The data that support the findings of this study are available from the corresponding author upon reasonable request.

## Code availability

Numerical PIC simulations were performed with the open source massively parallelized PIC code EPOCH, available at https://github.com/Warwick-Plasma/epoch. Data analysis and visualization was done with commercial software Matlab, Wolfram Mathematica and the open source visualization tool Visit, available at https://www.visitusers.org/index.php?title=Main_Page. All other relevant code used in this study is available from the corresponding author upon reasonable request.
